# Overcoming barriers to HPV vaccination in Pakistan: a critical need for a proactive and culturally-sensitive strategy

**DOI:** 10.1097/MS9.0000000000004398

**Published:** 2025-11-24

**Authors:** Syed Ahtisham Halim, Tirath Patel, Syeda Vilay Zehra Rizvi, Momina Khabir, Nikhilesh Anand

**Affiliations:** a Department of Medicine, Jinnah Sindh medical university, Karachi, Pakistan; b Department of Surgery, Trinity Medical Sciences University School of Medicine, Saint Vincent and the Grenadines; c Department of Medicine, Dow Medical College, Karachi, Pakistan; d Department of Medical Education, University of Texas Rio Grande Valley, Edinburg, TX, USA

**Keywords:** cervical cancer prevention, community engagement, cultural barriers, HPV vaccination, public health strategy

## Abstract

Cervical cancer continues to be one of the major causes of cancer-related mortalities among women worldwide, with a considerable share of this burden affecting developing countries like Pakistan. The addition of the HPV vaccine to the national immunization program of Pakistan had been a crucial leap toward the prevention of cervical cancer. Nevertheless, challenges that threaten the success of such a program are conspicuous. The most important ones are discussed in the letter, including the widespread dissemination of misinformation via social media on unfounded grounds, such as infertility resulting from the vaccine; deep-seated cultural taboos relating to sexual health; and an inert attitude of the government without a focused strategic communication. Mass psychogenic reactions, similar to the Tando Bago incident, further exacerbated the situation, along with disruptions in logistics due to natural calamities like floods. With this in mind, there is an imperative need for a multidimensional, proactive approach to ensure the long-term success of the HPV vaccination campaign. This should be in the form of public awareness campaigns adapted to culturally reorient attitudes, engagement of local community leaders and health providers, intensive vaccinator training, and incorporation of the vaccine into larger adolescent and reproductive health strategies. Public trust should be earned, equity assured, and, above all, the dramatic public health gains of the HPV vaccination assured through concerted efforts by government, civil society, and international partners.

*Dear Editor,*

Cervical cancer ranks as the fourth leading cause of cancer deaths among women globally^[1,2]^. It is the second most frequent cancer among women in low- and lower–middle-income countries^[3]^. According to the research, the distribution of cervical cancer differs across the world, with more than 85% of deaths occurring in developing regions^[4]^. Studies have shown a high association between human papillomavirus (HPV) and cervical neoplasia, despite the presence of additional risk factors^[5]^. Therefore, the prevention of HPV includes the primary vaccination of pre-adolescents and adolescents (girls between the ages of 9–18 years), as well as the early screening of cervical precancerous lesions such as CIN 3 and AIS in women over the age of 30 years through the use of HPV screening^[6]^. The American Cancer Society recently issued new recommendations on the use of HPV vaccinations in adults, strongly recommending that vaccinations begin as early as 9 years of age, compared to the traditional practice of initiating vaccinations at 11–12 years of age^[6]^. The update aims to enhance completion rates for the series and achieve optimal immunogenicity with earlier childhood vaccination. The ACS reconfirmed that individuals up to 26 years of age should be vaccinated or complete the entire course if previously unvaccinated, with specific recommendations for individuals initiating the course at later ages. However, beyond the age of 26 years, vaccinations are not recommended as current evidence shows limited benefit among this group^[7]^.

As of 15 September 2025, Pakistan had still not joined the ranks of the global community of nations that incorporate HPV vaccination into their national immunization initiatives. However, with the current national program to vaccinate girls between the ages of 9 and 14 years, Pakistan has now joined the over 150 countries, including Australia, the United Kingdom, the United States, and Rwanda, that successfully introduced HPV vaccination within the framework of their public health agenda^[8]^. Despite this historical introduction of the HPV vaccine into Pakistan’s national immunization program, the introduction to date has highlighted some crucial challenges. Misinformation spread throughout social media platforms, such as spurious allegations that the vaccine causes infertility, has led to hesitation among parents as well as flat refusals among families during the course of the program’s door-to-door outreach initiatives^[9]^. Despite this medium level of ground-level awareness of the availability of the vaccination, deeper levels of ignorance, as well as the cultural taboos around sexual health, persist with significant implications on the importance of culture-relevant education strategies^[10]^.

The government’s strategy to address the issues has been reactive and partial instead of strategically developed. There has been some development in vaccine acquisition and campaign implementation, but a straightforward, proactive approach to fighting misinformation, controlling public fears, or building cultural credibility is not evident. A reported case in Tando Bago, where 25 schoolgirls fainted after taking the cervical vaccine due to fear and possibly as a consequence of rumors or a compromised immune state, is one such example where the authorities did not use the opportunity to initiate campaigns of awareness or correct misconceptions, leaving room for misinformation as well as rumors to circulate in the absence of correct facts^[11]^. The situation of the floods across Pakistan has made things even harder to execute the HPV vaccination campaign. Extensive displacement, infrastructure destruction, and disruptions to health services made it harder to reach vaccination sites, particularly in rural regions and flood-affected areas^[12]^.

Despite the seriousness of these challenges, there has not yet been a proactive government response in the form of tailored strategies to ensure continued immunization during emergencies. The government must use a multipronged approach to ensure long-term success, including culture-aware awareness campaigns, community mobilization with local leaders and health providers, effective school- and community-centric delivery models, systematic training to build vaccinators’ capacity, and efficient safety and coverage monitoring systems. To put pressure on reluctance, increase equitable access, and achieve long-term public health benefits, the HPV vaccine must be included in the wider reproductive and adolescent health strategies, with an open, participatory approach involving civil society, UNICEF, WHO, and Gavi.

This letter adheres to the TITAN guideline for transparency in reporting the use of artificial intelligence in scientific writing^[13]^.

## Data Availability

No new dataset was generated during the study.

## Research registration unique identifying number (UIN)

Not required because the study is a letter to the editor.
